# Exploring the determinants of anemia in young adult women (20–24 years): the role of AI-based interventions in health management

**DOI:** 10.3389/frai.2026.1821975

**Published:** 2026-05-19

**Authors:** Sireesha Guttapalam, A. M. Beulah, M. Niharika, C. Pradeepthi, D. Madhavi

**Affiliations:** 1 Department of Home Science, Sri Padmavati Mahila Visvavidyalayam, Tirupati, Andhra Pradesh, India; 2 Department of CSE, SoET, Sri Padmavati Mahila Visvavidyalayam, Tirupati, Andhra Pradesh, India

**Keywords:** anemia, artificial intelligence, hematological parameters, machine learning and health, young adult women

## Abstract

**Background:**

Anemia is a deficiency disorder in healthy red blood cells or hemoglobin is a widespread health concern among young adult women, effects considerable impact on physical health, productivity, and overall quality of life. Despite its high prevalence, effective diagnosis and management remain challenging due to the complex interaction of the causes, intensified by limited awareness and inadequate health concern behavior.

**Objective:**

The present study aimed to study the determinants of anemia and development of AI App for screening anemia and management particularly for young women.

**Methods:**

A total of 545 women aged 20–24 years were examined in the study. A structured questionnaire was designed to acquire socio-demographic characteristics and to assess dietary and lifestyle factors influencing the probability of anemia risk. Blood samples were collected to evaluate key hematological parameters. Furthermore, an AI (artificial intelligence)-based anemia risk assessment and mobile health intervention was developed and effectiveness of the tool was evaluated using machine learning models.

**Results:**

Mild anemia constituted the largest proportion of the study population (50.3%), followed by normal hemoglobin status (27.5%). The machine learning model accuracy was 74.39% and human data diagnostic accuracy of the AI-based anemia assessment system was found to be 71%, respectively.

**Conclusion:**

This digital health approach also aims to enhance anemia awareness, prevention, and management. Integrating AI into anemia care holds promise for delivering accurate, timely, and personalized interventions, particularly in resource-restricted settings, thereby improving health outcomes for young women and reducing the societal burden of anemia.

## Introduction

Anemia is the most common deficient disorder in the world. There are lot of factors pays a critical role in its development, including deviations in the shape and size of the Red Blood Cells (RBC), insufficient production, breakdown, and loss of RBC, dysfunctional hemoglobin which can potentially cause anemia risk in adult females (Mwafy et al., 2022). During anemia, dysfunctional RBC can result in a wide range of symptoms from fatigue and weakness to severe and life-threatening conditions in chronic/prolonged periods. The most commonly practiced method for diagnosing anemia is a common blood test that includes a Complete Blood Count (CBC), hemoglobin concentration, hematocrit, and Mean Corpuscular Volume (MCV) (Hughes and Gill, 2026). However, in some cases or individuals, more advanced procedures have been required to differentiate between types of anemia. Worldwide, it was considered as a major public/societal health problem that affected billions of people. Nearly 1.8 billion people affected until 2019 (Ghosal et al., 2023). The actual prevalence in 2023 is slightly higher but similar, showing a slow progressive trend. Coming to India, anemia is highly prevalent, where the National Family Health Survey (NFHS) reported that 57% of women aged 15–49 years and 67% of children under 5 years of age were suffering from anemia (Girotra et al., 2023).

In India, anemia is a serious societal mass health concern, placing second among the causes of maternal mortality with a higher prevalence than many other developing countries. In spite of increased national and international (WHO) attention and preventive actions by the government and non-governmental organizations, prevalence of anemia has increased in the Indian population to nearly 45% since 1990 which is closely associated with iron deficiency (Natekar et al., 2022). A study by Zamani et al., 2025, described the several risk factors associated with iron deficiency anemia have been identified which include dietary, socioeconomic, and genetic factors. Iron deficiency anemia is associated with unusual health outcomes like fatigue, poor mental health, and difficulties conceiving/giving birth. However, appropriate and cost-effective interventions can help prevent these effects (Zamani et al., 2025). The 5th NFHS has significantly highlighted and pinned the need of addressing the concerns of anemia in the society, especially in rural and societally disadvantaged communities where it is most prevalent. The NFHS is strategically providing key statistical information on anemia occurrence and prevalence, but changes in diagnostic procedures and consequent exclusions from the program have raised concerns about the integrity and accuracy/reliability of the data.

The prevalence of anemia among Indian women aged 15–49 is projected to decrease between 2024 and 2028, from 52.8% in 2024 to 52.6% in 2028, and monitoring socioeconomic factors and access to healthcare services is important to identify and control this trend (Forecast: Prevalence of Anemia in India, 2024). The World Health Organization’s (WHO) global nutrition targets aim to reduce the prevalence of anemia among women of reproductive age (15–49 years) by 50% by 2030 (WHO/UNICEF, 2019).

During recent years the higher risk for anemia has increased among university students, especially in developed countries due to improper intake of poor nutrition and due to insufficient healthcare access (Hamali et al., 2020). The IDA (Iron Deficiency Anemia) was also due to inadequate intake of Iron rich and B12 rich foods (Bathla and Arora, 2022). In addition the students of universities are having undesirable food choices, with high intake of fast foods and low consumption of fresh fruits and vegetables and also due to economic restrictions or shortage of time to cook meals.

Anemia is a clinical condition in which if not treated there will be not enough healthy red blood cells in the blood, leading to reduced oxygen delivery to various organs in the body. Iron deficiency anemia develops due to inadequate iron intake. Without enough iron, the body cannot produce enough hemoglobin, an essential component of red blood cells, which impairs oxygen delivery.

Iron deficiency in human bodies can be due to various etiological factors. The cause can be a disorder of any physiological system, particularly a malfunctioning digestive system. Due to insufficient iron intake, a deficiency of hemoglobin can occur. Because of this, the bone marrow is also unable to produce enough hemoglobin. Most cases of iron deficiency anemia are mild and without complications. This condition is usually easily correctable. However, if anemia or iron deficiency is left untreated, it can lead to other health problems (Paul et al., 2021).

The “Anemia Mukt Bharat” campaign, launched in 2019, was intended to reduce or eliminate the problem of anemia, but data from the NFHS 5 shows a significant increase in anemia cases among women of all ages over the past 5 years. Therefore, the programs need to be reviewed and made more effective (Maji et al., 2023).

According to the WHO guidelines on daily iron intake for adult women and adolescents, a preventive dose of 30–60 mg of elemental iron in tablet form, daily for three consecutive months of the year, is recommended for non-pregnant women of reproductive age in countries with an anemia prevalence of 40% (Anemia in Women and Children, 2023). The WHO has the global goal of reducing anemia by 50% in women of reproductive age by 2025 (Kalaivani and Ramachandran, 2025).

Artificial intelligence (AI) has emerged as an advanced technology which mimics human-like intelligence and executes tasks (normally requiring human cognitive inputs) without human cognitive interference (Haghi et al., 2023). AI enabled models/programs are able to understand the patterns, and relationships and can identify and analyze images/data. In the case of anemia disorder, the studies demonstrated and explained that AI enabled algorithms can analyze images of various physical characteristic features like conjunctiva, palms, fingernails and tongue. Moreover, by assessing hemoglobin concentration, these algorithms can also predict the anemia in the masses. Furthermore, AI driven systems can analyze acquired clinical data including laboratory tests and blood tests to predict the occurrence and type of anemia. Also, these AI enabled programs can accurately analyze clinical symptoms and laboratory parameters and create a more affordable and accessible way to diagnose anemia with reasonable reliability (Ferih et al., 2023).

Hence, there is a need to advance a mobile based application which helps in assessing anemia status using a robust technology of the young adult women population.

The significance of the study includes the consequences for young adult women where anemia can lead to fatigue, difficulty in concentrating and reduced work productivity. Anemia during pregnancy can increase the risk of maternal mortality, low birth weight and preterm birth. It weakens the immune system making individuals more vulnerable to infections. It negatively impacts overall quality of life, affecting social, emotional and psychological wellbeing.

The objectives of the present study include identifying the prevalence of anemia among young adult women in the target population. Determining the socio demographic factors associated with anemia (age, education, occupation, income, marital status and residence). Assess the dietary factors contributing to anemia. Evaluating the lifestyle factors influencing the anemia condition. Developing and validating an AI based model to predict anemia risk based on identified determinants. Design and implement an AI powered mobile health intervention to improve anemia awareness, prevention and management and finally evaluate the effectiveness of the AI based intervention in reducing anemia prevalence and improving health outcomes.

## Methodology

### Study design

**Figure 1:**
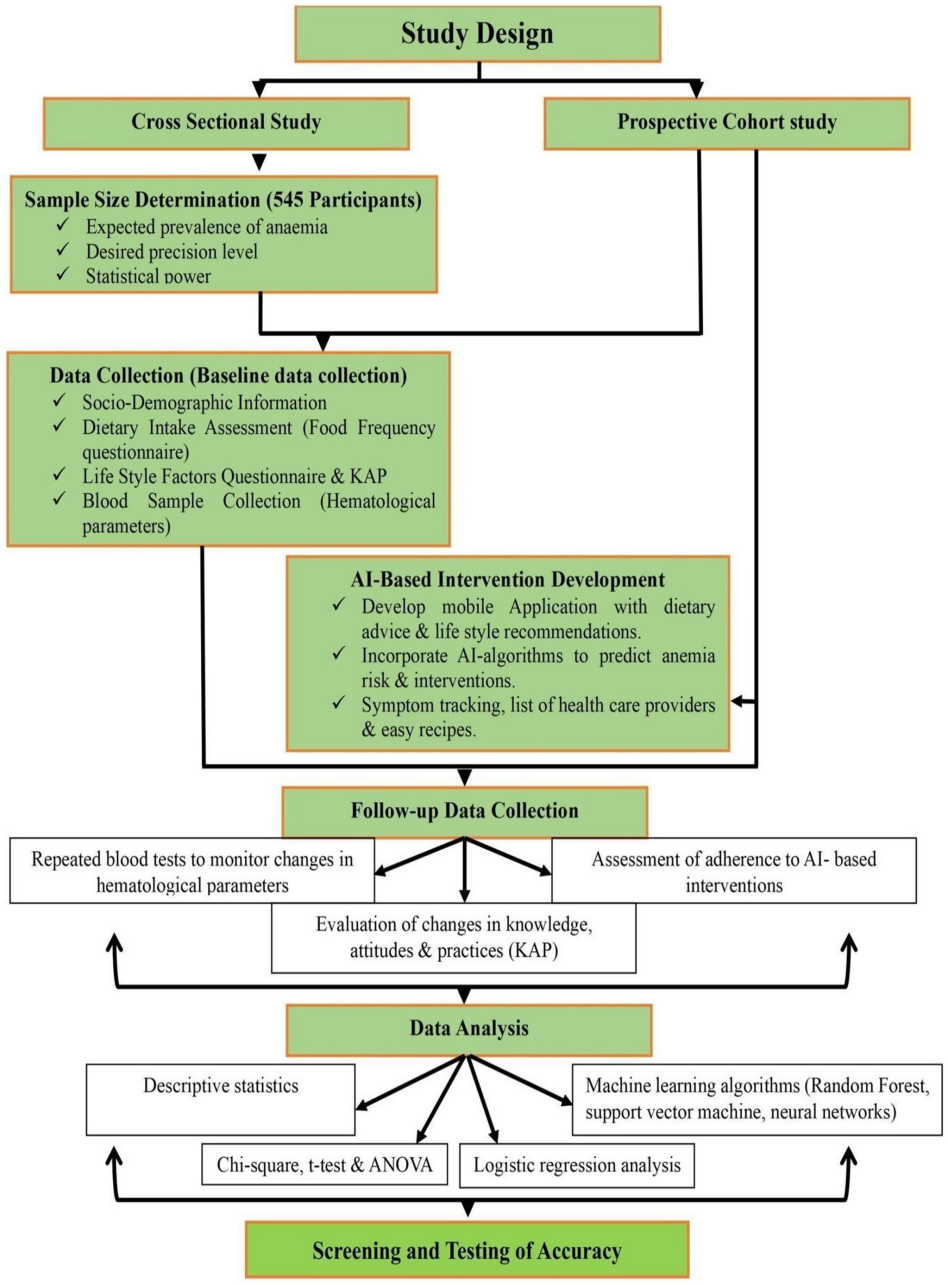
Experimental design of the study.

The present study adopted a diversified-method research method comprising a cross-sectional analytical study followed by a prospective design to systematically assess anemia among young adult women and to evaluate the effectiveness of an AI-based intervention. Figure 1 shows the experimental design of the study. The cross-sectional element was used to estimate the occurrence of anemia and to identify its socio-demographic, dietary, and lifestyle factors, and for examining changes for anemia status the prospective cohort was conducted where AI-based mobile health intervention was implemented for following outcomes.

### Sampling strategy

The study was piloted among young adult women aged 20–24 years residing in Tirupati, Andhra Pradesh. A cross sectional survey was conducted to determine the prevalence rate of anemia and determinants of anemia. The participants from this survey were mild and moderate anemic persons randomly selected for prospective cohort study.

### Sample size calculation

The sample size was calculated based on a supposed occurrence of anemia, a confidence level of 95%, and a margin of error of 5%.

The minimum sample size is calculated using the equation: $\frac{N=Z^{2}\times \mathrm{PX}(1-P)\;}{d^{2}}$

Z-score (standard normal deviate), which is 1.96 for a 95% confidence level. Estimated prevalence of anemia (in decimal form). For 60%, use 0.60.

Absolute precision (margin of error), typically 5% (0.05).

Accordingly, a sample size of 545 participants was determined to be adequate to safeguard enough statistical power and reliable valuation of anemia commonness in the target population. Sri Padmavati Mahila Visvavidyalayam, Sri Venkateswara University and Sri Venkateswara College of Engineering were selected for the study because young adults were studying in these institutions from different parts of Andhra Pradesh.

*Inclusion criteria* for participants were young adult woman students’ age group between 20 and 24 years, both hostellers and day scholars,’ students of Science, Technology and Humanities. The students who are willing to participate in the study; Informed consent was taken from each subject. Suitable participants were employed using appropriate sampling techniques after obtaining written informed consent. *Exclusion criteria* for study subjects who are not willing, pregnant, lactation and severe health issues.

Baseline data of anemic and non-anemic young adults’ were collected using a structured and pre-tested questionnaire with the inclusion of socio-demographic information such as age, education, occupation, marital status, residence, and family income and other relevant factors were collected. Dietary intake was evaluated using a food frequency survey designed to assess the habitual intake of iron-rich foods, folate-containing foods and vitamin B12 and also includes dietary practices that influence iron absorption. Lifestyle aspects including physical activity, eating practices; anxiety levels, sleep patterns, smoking, and liquor consumption were evaluated using a questionnaire. Therefore venous blood samples were collected under sterile conditions by qualified persons to estimate hematological factors, together with hemoglobin concentration, hematocrit, and mean corpuscular volume, to regulate anemia status.

World Health Organization (WHO) states that certain hemoglobin thresholds are used to diagnose anemia (World Health Organization, 2024). Normal Hb levels >12 g/dL; Mild anemia by Hb levels of 11.0 to 11.9 g/dL in non-pregnant women and 10.0 to 10.9 g/dL in pregnant women; Moderate anemia by Hb levels of >8.0–10.9 g/dL in non-pregnant women and 7.0 to 9.9 g/dL in pregnant women; Severe anemia by hemoglobin levels falling below <8.0 g/dL in non-pregnant women and below 7.0 g/dL in pregnant women (Sappani et al., 2023).

The timeline of the present study for the development and standardization of questionnaires from March to April 2025. Data collection (baseline data, socio-demographic, food frequency and lifestyle factors) and hematological parameters (Hemoglobin, Hematocrit and MCV) assessment of anemia prevalence from May to July 2025. Data pooling and analysis, identification of determinants of anemia in the month of August 2025. Development of intervention module and pilot testing, AI tool development from the month of September to November 2025. Intervention from December 2025 to January 2026 and outcome measurements were collected during February 2026.

### Intervention description

After following baseline assessment, participants who were recognized as eligible for intervention were included in the prospective cohort phase. For the intervention program 50 members from mild and 50 members from moderate anemic subjects were randomly selected; except blood parameters remaining all parameters were assessed and compared after intervention. AI-based mobile health intervention app was developed and implemented which includes AI-driven anemia risk assessment, tailored dietary recommendations, custom-made meal plans, suggestion of iron-rich recipes, lifestyle modification guidance, and informative content related to anemia prevention and management. Machine learning algorithms have been explored to predict anemia risk based on individual-level data, including dietary habits, lifestyle aspects, and hematological indicators, and to create personalized health recommendations.

Participants received orientation and training regarding the usage of the mobile app, and constant provision was delivered throughout the intervention period. The application was accessible as both a mobile app and a web app. The system was developed as a Progressive Web App (PWA). The PWA could be downloaded and installed as a mobile app across Android, iOS, and ChromeOS, and was also used as a website on desktops and laptops. To assess changes in individual hematological parameters like repeated blood tests were done and follow up data was also collected in a prescribed interval period. Adherence to the intervention was assessed using both self-reported adherence questionnaires and spontaneously generated app usage data, including usage frequency, period of engagement, and task end. Validated tools like Knowledge, attitude, and practice were used to measure progress in awareness, behavior changes, and implementation of health and well practices were done related to anemia.

### Statistical analysis

Potential confounding variables were found in this investigation based on the relevance of the study and the quantity of existing literature. Age, gender, socioeconomic status, and lifestyle elements like food consumption, exercise, and personal hygiene were among them. Hematological estimate from blood samples and structured general and lifestyle questionnaires were used to gather information on these variables.

Anemia status was used as the dependent variable in multivariable logistic regression analysis to account for confounding. The results were shown as adjusted odds ratios with 95% confidence intervals after all identified variables were incorporated into the model to get adjusted estimates. These characteristics were also integrated by the AI-based application to improve forecast accuracy and facilitate data processing.

Data analysis was carried out by using suitable statistical and machine learning techniques. To summarize socio-demographic features, lifestyle factors, dietary patterns and anemia prevalence descriptive statistics was used. Inferential statistical tests such as chi-square tests, independent *t*-tests, and analysis of variance (ANOVA) were applied to compare anemic and non-anemic groups. To identify significant determinants of anemia, Logistic regression analysis was performed. In the context of anemia, several Machine learning algorithms such as Random forest, logistic regression, support vector machine, neural networks, decision tree and XGBoost have been utilized regarding anemia diagnosis For this study Ethical approval was acquired from the Institutional Ethics Committee (IEC), Sri Padmavati Mahila Visvavidyalayam, (Women’s University, Tirupati, Andhra Pradesh, India, The IEC No. SPMVV/Acad/IEC/C1/VIII/2025, dt:27-03-2025). Written informed consent was obtained from all participants earlier to data collection. All the procedures were conducted in accordance with ethical guidelines for human research and throughout the study the participant information was strictly kept confidential.

### Machine learning methodology steps

#### Data preprocessing

The dataset consisted of demographic, clinical, dietary, and lifestyle variables. Irrelevant or leakage-prone features were removed to prevent model bias. All categorical variables were converted to uppercase to maintain consistency. Missing categorical values were handled by imputing “Unknown.” Categorical variables were encoded using Label Encoding. The target variable anemic classification was binarized: 0 → Normal, 1 → Anemic (mild/moderate/severe).

#### Feature selection

Two complementary techniques were used: 1. Mutual Information (MI): Measures dependency between features and target. 2. Random Forest Feature Importance: Based on Gini importance. A combined ranking strategy was used: Features were ranked using both methods. An average rank was computed. Top N features (experimentally optimized) were selected.

#### Dataset splitting

The dataset was split into: 1. 70% Training set, 2. 30% Testing set. Stratified sampling was used to maintain class balance: train_test_split (…, stratify = y).

#### Model training

Multiple machine learning models were trained: 1. Random Forest (various configurations), 2. Logistic Regression (including balanced), 3. Support Vector Machine (RBF kernel), 3. K-Nearest Neighbors, 4. Decision Tree, 5. XGBoost.

#### Special handling

Class imbalance addressed using: class weight = “balanced.”

#### Model selection

Different feature sizes (*N* = 20, 25, 28, etc.) were tested. Best performance achieved with: Random Forest (Balanced) and Top 25 features.

AI model is developed using RandomForestClassifier and compiled as a PKL file and wrapped around a Flask backend to be exposed as a REST API to be invoked from our PWA. Risk assessment tool is developed as a PWA—which takes the input from user and forwards the user input to our backend developed using Flask. Using the factors given by user-risk is assessed for anemia and result is displayed back to the User in the Web App or Mobile App.

### Testing of App accuracy

The AI application developed on *anemia-spmvv.pages.dev* is a non-invasive, smartphone-based tool designed to assist in the early screening of anemia by utilizing artificial intelligence. This web-based system analyzes input data, such as user hemoglobin levels or other relevant features, and classifies individuals into categories like normal, mild, moderate, and severe anemia with real-time feedback. The primary aim of the app is to provide a rapid, accessible, and user-friendly preliminary assessment that can support routine health monitoring, especially in community or low-resource settings where laboratory testing may be limited. By leveraging machine learning algorithms, the application enhances efficiency in identifying at-risk individuals and empowers users with actionable insights, while emphasizing that clinical confirmation remains essential for definitive diagnosis.

The multiple algorithms were trained and compared, with the best model achieving an accuracy of 74.39%. Feature selection was performed using mutual information and random forest importance ranking, and SMOTE was applied to handle class imbalance. An example of scoring is that the model takes input features (e.g., fatigue level, iron intake, stress level, sleep quality) and outputs probability scores for each anemia class, selecting the most probable class as prediction.

Out of 107 AI assessments compared with the original hemoglobin classification, 76 cases were correctly classified and 31 cases were misclassified. The overall diagnostic accuracy of the AI-based anemia assessment system was found to be 71%. Most errors occurred when the AI labeled participants as moderate or severe anemia while the original hemoglobin levels indicated normal or mild anemia. The AI showed better consistency in detecting clear cases of anemia but had difficulty accurately identifying normal individuals.

### Model performance evaluation

The performance of the machine learning models was evaluated using multiple metrics, including accuracy, precision, recall, F1-score, and ROC-AUC. The best-performing model was the Random Forest classifier with class balancing, trained on the top 25 selected features. The model achieved: Accuracy: 74.39%, Precision (Anemic class): 0.74, Recall (Anemic class): 0.99, F1-score (Anemic class): 0.85, ROC-AUC Score: 0.53. The confusion matrix analysis revealed that the model demonstrates very high sensitivity (recall) for detecting anemia but relatively poor specificity for identifying non-anemic individuals.

The AI program developed is a machine learning based anemia classification system that predicts four categories (Normal, mild, moderate,severe) using health,lifestyle,dietary and symptom related features. The present analysis demonstrates that the AI-based anemia assessment tool achieved a moderate level of agreement with laboratory hemoglobin classification, with an overall accuracy of 71%. The model performed relatively well in identifying individuals with anemia, particularly in severe cases where hemoglobin levels were clearly low. However, it showed limitations in distinguishing between normal and borderline categories, which contributed to false-positive classifications. This suggests that while the AI system may serve as a useful preliminary screening tool in community settings, it should not replace laboratory confirmation. Further refinement of the algorithm, inclusion of additional clinical parameters, and expansion of the dataset may improve its specificity and overall diagnostic performance.

After development of the App, App was shared with the participants and instructed them to follow the App guidelines on anemia—causes, types, symptoms and management, after that again KAP of the subjects were assessed.

## Results

The demographic profile of the 545 participants revealed a predominance of young adults, with the majority aged 21 years (41.28%), followed by those aged 20 years (25.32%) and 22 years (22.20%) was presented in Table 1. Participants aged 23 and 24 years constituted smaller proportions of the study population. With respect to marital status, most respondents were unmarried (97.8%), reflecting the youthful composition of the cohort, while only small fractions were married (2.2%). With 50.46% residing in urban and remaining 49.54% in rural settings, it represents both environments. 76.51% of participants were hostellers, whereas day scholars accounted for 23.49%. Blood group analysis showed that B + (35.05%) and O + (32.84%) were the most prevalent blood groups among participants. A + (11.01%) and AB+ (6.61%) were less common, while negative blood groups such as O-, B-, A-, and AB-together comprised a relatively small proportion of the sample.

**Table 1:** Demographic characteristics of the study participants (*n* = 545).

Demographic profile		
Parameter	Count	Percentage (%)
Age (years)		
21	225	41.28
20	138	25.32
22	121	22.2
23	39	7.16
24	22	4.04
Marital status		
Unmarried	533	97.8
Married	12	2.2
Residence		
Urban	275	50.46
Rural	270	49.54
Blood group		
B+	191	35.05
O+	179	32.84
A+	60	11.01
AB+	36	6.61
O−	24	4.4
B−	22	4.04
A−	22	4.04
AB−	11	2.02
Stay		
Hosteller	417	76.51
Day scholars	128	23.49

**Figure 2:**
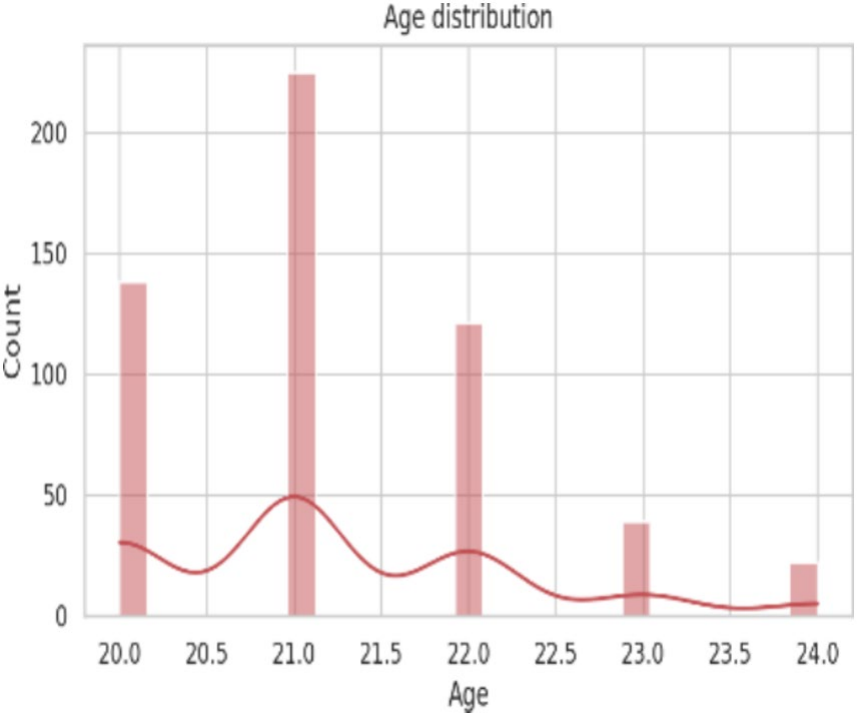
Age wise distribution of the subjects.

Table 2 presents the distribution of anemia severity across demographic characteristics. From 545 subject’s different age groups, mild anemia was the most prevalent category across all ages 20–22 years particularly. The remaining half of the subjects was mildly anemic. One-fifth of participants observed moderate anemia in these age groups, while less than 6% across all age groups are affected with severe anemia (Figure 2). 50.84% belonged to unmarried and exhibited a similar pattern, with mild anemia being most frequent (50.84%), followed by moderate anemia (17.82%). Married participants showed a comparatively higher proportion of moderate anemia (41.67%), although their overall number was small.

**Table 2:** Anemia severity based on the demographic characteristics of the subjects.

Demographic	Frequency (percentage)				Total
	Normal	Mild	Moderate	Severe	
Age (years)					
20	34 (24.64)	70 (50.72)	26 (18.84)	8 (5.8)	138
21	66 (29.33)	109 (48.44)	45 (20)	5 (2.22)	225
22	31 (25.62)	59 (48.76)	24 (19.83)	7 (5.79)	121
23	12 (30.77)	25 (64.1)	2 (5.13)		39
24	7 (31.82)	11 (50)	3 (13.64)	1 (4.55)	22
Marital status					
Married	3 (25)	3 (25)	5 (41.67)	1 (8.33)	12
Unmarried	147 (27.58)	271 (50.84)	95 (17.82)	20 (3.75)	533
Residence					
Rural	77 (28.52)	137(50.74)	46 (17.04)	10 (3.7)	270
Urban	73 (26.55)	137 (49.82)	54 (19.64)	11 (4)	275
Stay					
Day scholars	41 (32.03)	60 (46.88)	23 (17.97)	4 (3.12)	128
Hosteller	109 (26.14)	214 (51.32)	77 (18.47)	17 (4.08)	417
Blood group					
A+	17 (28.33)	36 (60)	5 (8.33)	2 (3.33)	60
A−	4 (18.18)	12 (54.55)	6 (27.27)		22
AB+	3 (8.33)	19 (52.78)	10 (27.78)	4 (11.11)	36
AB−	2 (18.18)	6 (54.55)	3 (27.27)		11
B+	50 (26.18)	99 (51.83)	34 (17.8)	8(4.19)	191
B−	8(36.36)	8 (36.36)	5 (22.73)	1(4.55)	22
O+	61(34.08)	83 (46.37)	30 (16.76)	5 (2.79)	179
O−	5 (20.83)	11 (45.83)	7 (29.17)	1(4.17)	24

Mild anemia affected approximately half of the participants in both settings of rural and urban areas, while moderate anemia was slightly more common among urban residents. Severe anemia remained low in both groups. 51.32% hostellers had a higher prevalence of mild and 18.47% moderate anemia compared to day scholars. Severe anemia was marginally higher among hostellers (4.08%) than day scholars (3.12%). According to blood group wise distribution mild anemia was predominant across all blood groups. Notably, AB+ blood group showed a relatively higher proportion of moderate (27.78%) and severe anemia (11.11%) compared to other groups.

Bivariate analysis showed statistically significant association between AB+ blood group and anemia (COR = 4.349; 95% CI: 1.175–16.094; *p* = 0.0277). Other variables, including age, marital status, residence, and stay, did not show statistically significant associations. Multivariable logistic regression analysis confirmed that participants with AB+ blood group had significantly higher odds of anemia even after adjusting for confounders (AOR = 4.615; 95% CI: 1.239–17.196; *p* = 0.0227). No other demographic variables were independently associated with anemia.

**Figure 3:**
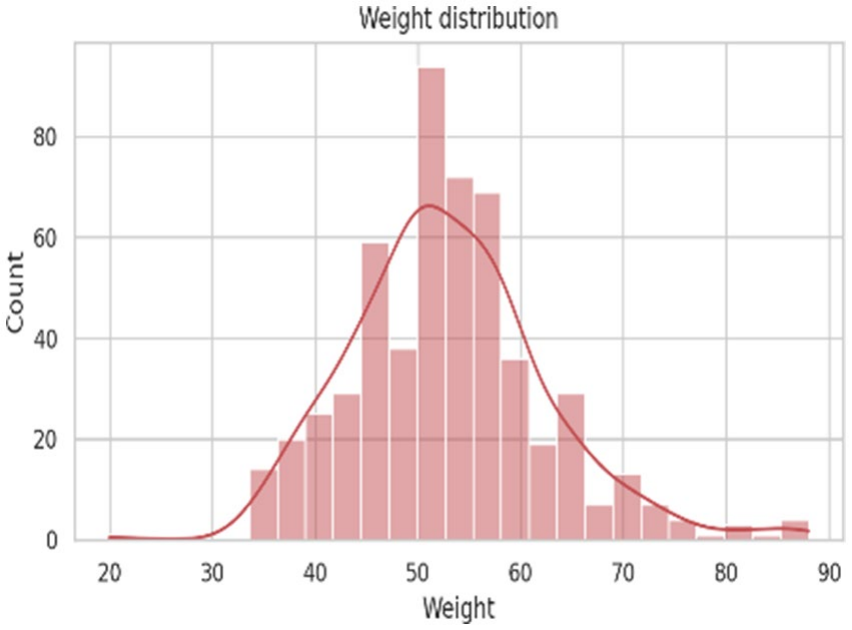
Weight distribution of the study subjects.

**Figure 4:**
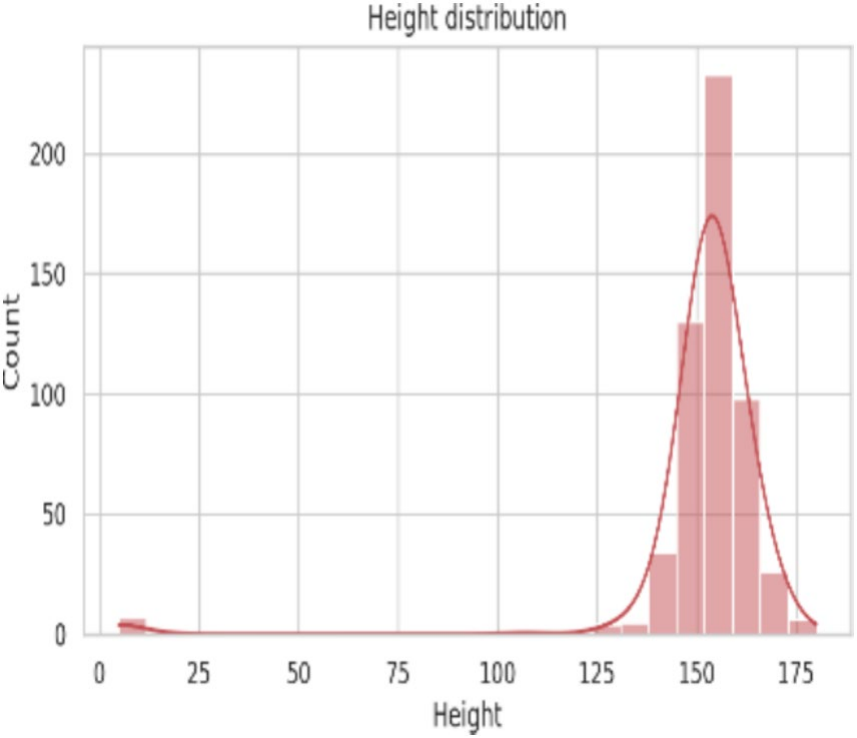
Height distribution of the study subjects.

The descriptive statistics of demographic, lifestyle, and hematological parameters of the 545 study participants are presented in Table 3. The mean age (Figure 2) of the participants was 21.23 ± 1.03 years, with an age range of 20 to 24 years, indicating a relatively homogeneous young adult population. The average body weight (Figure 3) was 52.87 ± 9.49 kg, while the mean height (Figure 4) was 152.00 ± 19.37 cm. Lifestyle-related variables showed moderate self-reported scores, with mean energy level and sleep quality scores of 3.19 ± 0.84 and 3.51 ± 0.91, respectively, on a five-point scale.

**Table 3:** Descriptive statistics of demographic, lifestyle and hematological parameters.

Variable	Mean	Standard deviation (SD)
Age (years)	21.23	1.04
Weight (kg)	52.87	9.49
Height (cm)	152	19.37
Energy Level	3.19	0.84
Sleep Quality	3.51	0.91
Hemoglobin (gms %)	11.02	1.53
Total WBC Count (cells/cumm)	8074.02	2122.85
RBC Count (m/cumm)	12.41	184.01
Neutrophils (%)	58.11	7.73
Lymphocytes (%)	34.32	7.2
Eosinophils (%)	3.21	0.85
Monocytes (%)	4.38	1.34
Basophils (%)	0	0
Platelet Count (lakhs/cumm)	3.06	0.7
Packed Cell Volume (PCV) (%)	36.44	4.45
MCV (fL)	82.32	13.19
MCH (pg)	25.35	9.28
MCHC (%)	30.07	2.04

**Figure 5:**
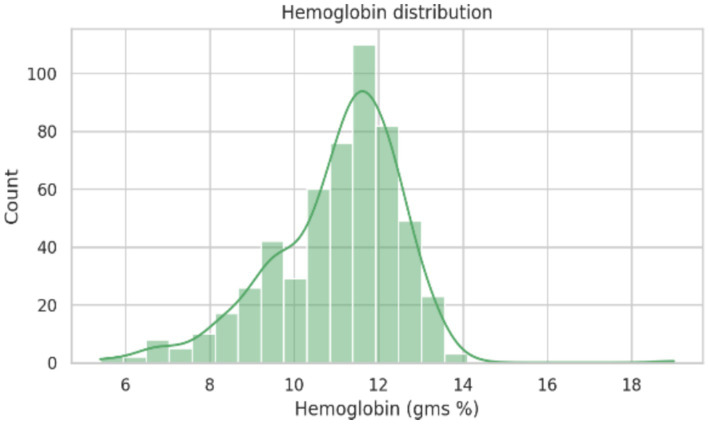
Overall distribution of hemoglobin concentration among participants.

Hematological assessment revealed a mean hemoglobin concentration (Figure 5) of 11.02 ± 1.53 g/dL, with values ranging from 5.4 to 19 g/dL, it indicates the presence of varying degrees of anemia within the study population. The mean total white blood cell count was 8,074 ± 2,123 cells/cumm, falling within the expected physiological range. Red blood cell indices demonstrated a mean packed cell volume (PCV) of 36.44 ± 4.45%, mean corpuscular volume (MCV) of 82.32 ± 13.19 fL, mean corpuscular hemoglobin (MCH) of 25.35 ± 9.28 pg., and mean corpuscular hemoglobin concentration (MCHC) of 30.07 ± 2.04%.

Differential leukocyte counts showed neutrophils constituting the largest proportion (58.11 ± 7.73%), followed by lymphocytes (34.32 ± 7.20%). Platelet counts averaged 3.06 ± 0.70 lakhs/cumm, with most values remaining within normal limits. Overall, the hematological profile indicates mild alterations in red cell parameters, consistent with nutritional or early-stage anemia patterns in young adults.

A comparison between normal and anemic persons revealed a distinct physiological, lifestyle, and dietary patterns. Hematological indices were significantly reduced in the anemic group, with lower mean hemoglobin (10.42 g/dL vs. 12.60 g/dL), Packed cell volume (34.82% vs. 40.71%), MCHC values confirming compromised red cell mass and oxygen transport.

**Figure 6:**
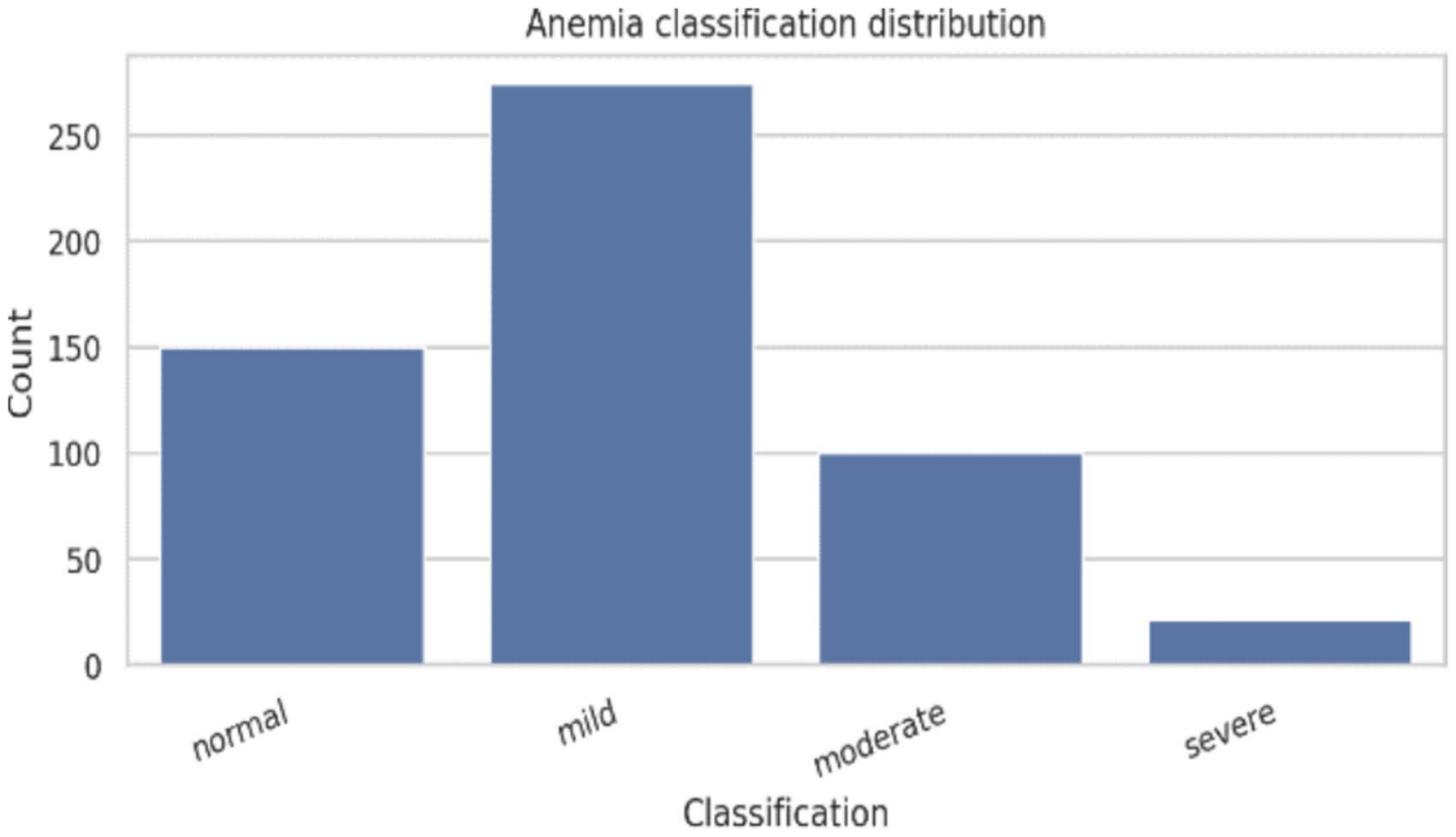
Distribution of anemia severity classification in the study population.

**Figure 7:**
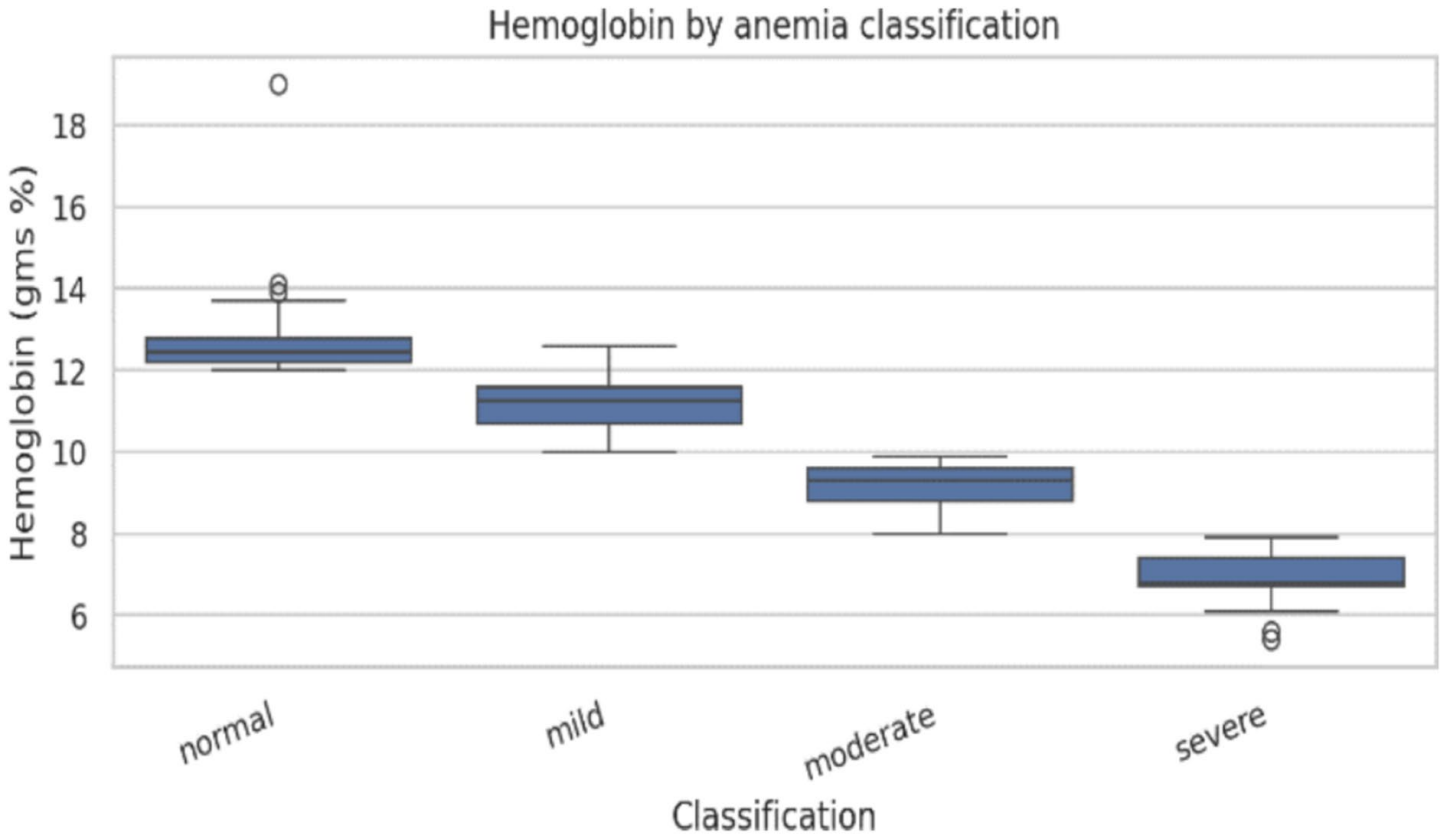
Hemoglobin levels across anemia severity categories.

Anthropometric assessment showed a near-normal distribution of height and weight (Figures 3, 4), with most participants falling within expected ranges for young adults, suggesting the absence of extreme under nutrition or obesity at the population level. Despite this, hemoglobin analysis revealed a substantial burden of anemia. The hemoglobin distribution demonstrated a leftward shift, with values spanning from severe deficiency to normal levels (Figures 5–7).

**Figure 8:**
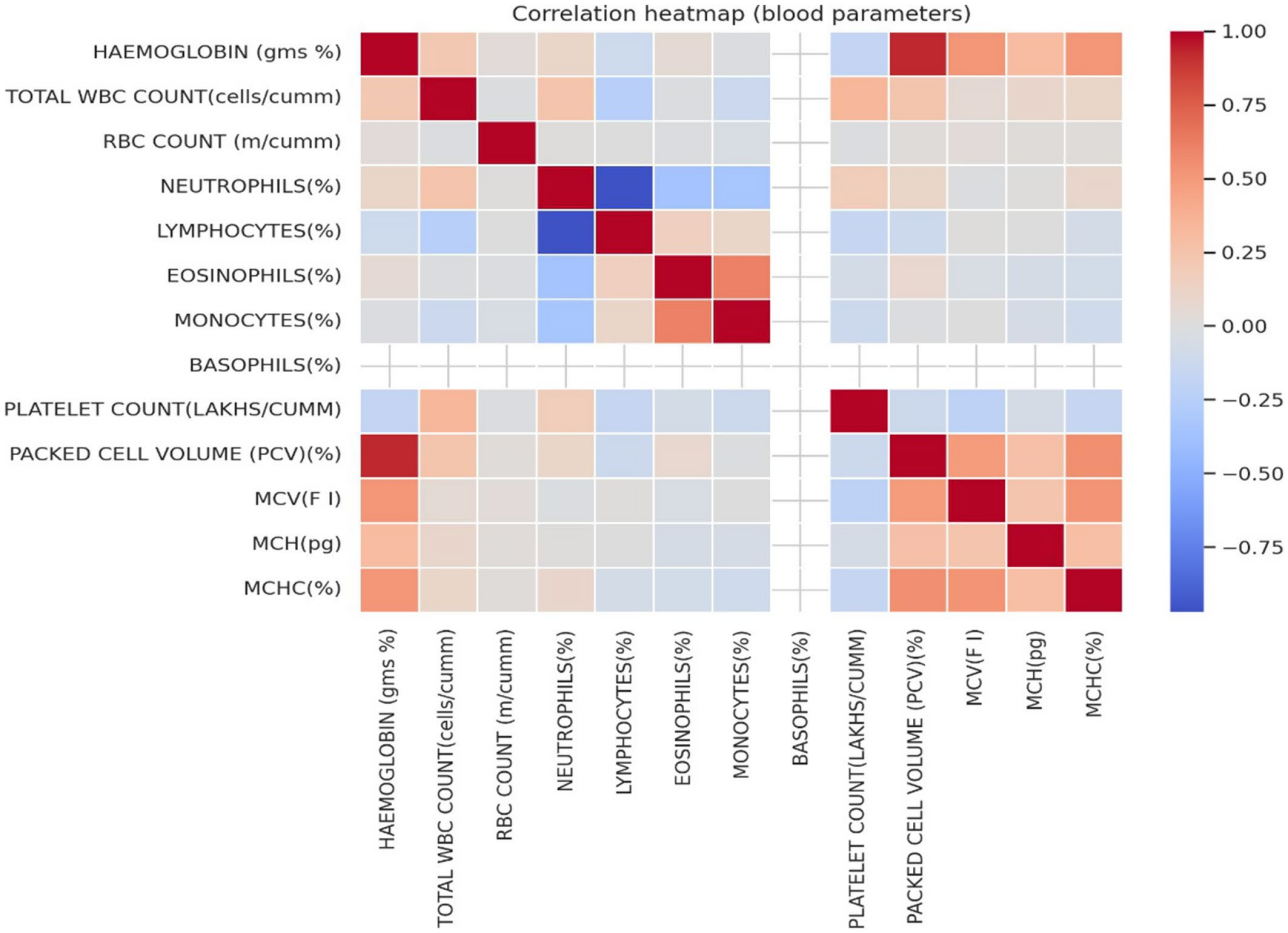
Correlation heat map illustrates relationships among hematological parameters, including hemoglobin, red blood cell indices, white blood cell differentials, and platelet count.

**Figure 9:**
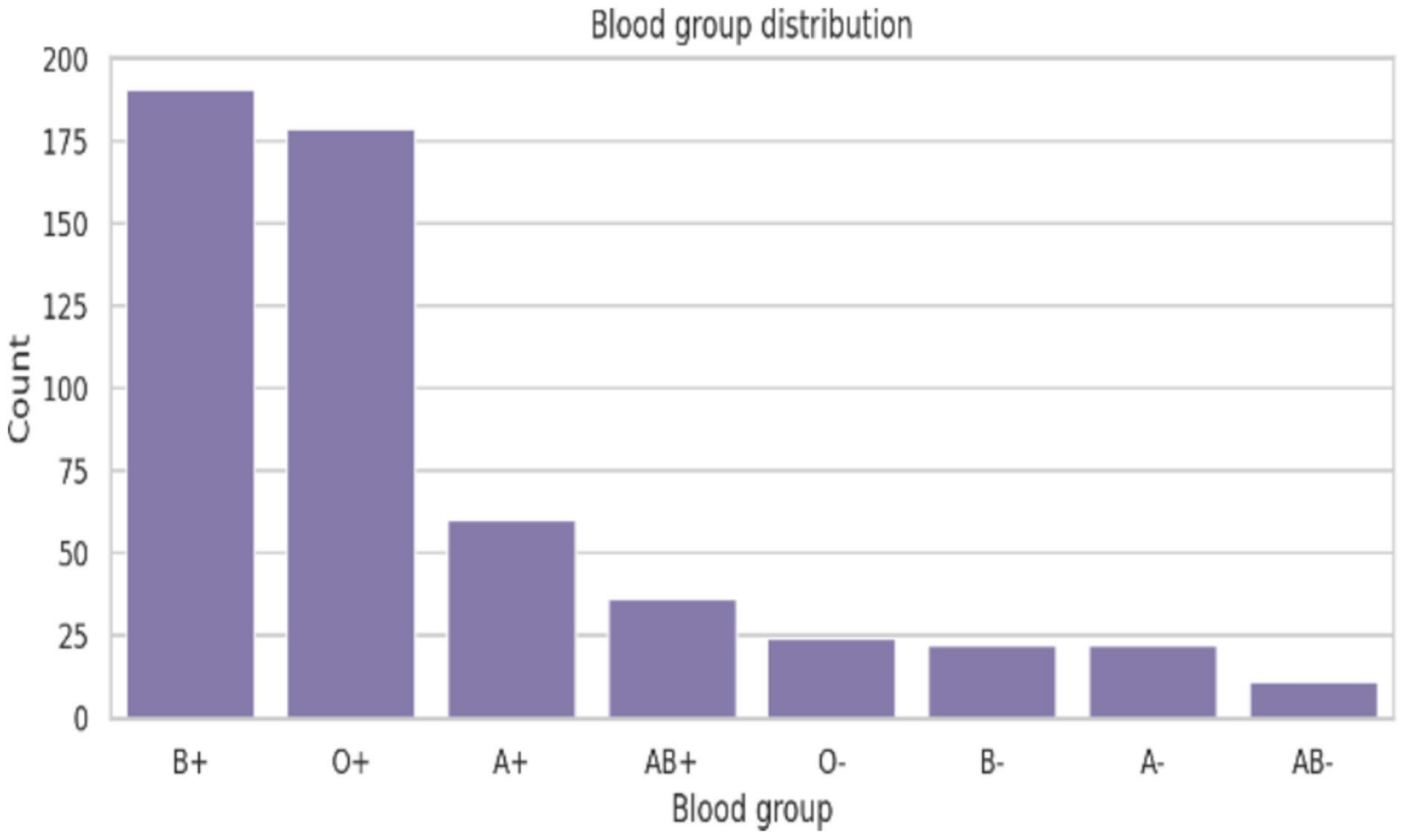
Distribution of ABO and Rh blood groups among the participants.

Correlation analysis of hematological parameters in Figure 8 indicated strong positive associations between hemoglobin, packed cell volume (PCV), mean corpuscular volume (MCV), mean corpuscular hemoglobin (MCH), and mean corpuscular hemoglobin concentration (MCHC). In contrast, inflammatory cell markers such as neutrophils and lymphocytes showed weaker or inverse relationships with red cell indices. Platelet count demonstrated a modest inverse association with hemoglobin and PCV. Blood group distribution in Figure 9 revealed predominance of B + and O + groups, while Rh-negative groups were relatively uncommon.

The distribution of participants according to anemia severity presented in Table 4 revealed that mild anemia constituted the largest proportion of the study population (50.3%), followed by normal hemoglobin status (27.5%). Moderate anemia was observed in 18.3%, while severe anemia affected a smaller fraction (3.9%) of participants. The progressive increase in the frequency and intensity of reported clinical signs and symptoms such as fatigue, generalized weakness, pallor, reduced exercise tolerance, and dizziness was evident with increasing anemia severity. Participants classified under moderate and severe anemia categories demonstrated a substantially higher symptom burden compared to those with normal hemoglobin levels. Statistical analysis showed a significant association between anemia severity and the presence of clinical symptoms (*χ*^2^ test, *p <* 0.05), indicating that symptom manifestation escalates in parallel with declining hemoglobin concentration.

**Table 4:** Distribution of common signs and symptoms according to anemia severity.

Signs and symptoms	Normal *n* (%)	Mild *n* (%)	Moderate *n* (%)	Severe *n* (%)	χ^2^ value	*p*-value
Fatigue	42 (28.0)	168 (61.3)	82 (82.0)	20 (95.2)	64.21	<0.001*
Pallor	18 (12.0)	124 (45.3)	76 (76.0)	19 (90.5)	88.47	<0.001*
Dizziness	21 (14.0)	98 (35.8)	61 (61.0)	17 (81.0)	59.36	<0.001*
Headache	26 (17.3)	113 (41.2)	54 (54.0)	14 (66.7)	34.18	<0.001*
Shortness of breath	9 (6.0)	64 (23.4)	49 (49.0)	16 (76.2)	91.02	<0.001*
Palpitations	7 (4.7)	52 (19.0)	41 (41.0)	15 (71.4)	86.55	<0.001*

*Statistically significant at *p* < 0.05 (Chi-square test).

A progressive increase in the prevalence of clinical signs and symptoms was observed with worsening anemia severity. Fatigue emerged as the most frequently reported symptom, affecting 61.3% of individuals with mild anemia and increasing sharply to 95.2% among those with severe anemia. Pallor and dizziness also showed a marked rise from normal hemoglobin status to severe anemia, with pallor present in over three-quarters of participants with moderate anemia and more than 90% of those with severe anemia.

Symptomatically, anemic individuals reported slightly higher frequencies of headaches, cognitive difficulty, and un-refreshed sleep, alongside higher perceived stress levels. From a dietary perspective, the anemic group demonstrated lower meat and vitamin C intake and a higher proportion of vegetarian participants, consistent with reduced heme-iron availability and impaired non-heme iron absorption. These differences collectively align with known clinical drivers of iron-deficiency anemia and support the inclusion of dietary, symptom, and lifestyle features in screening and risk stratification tools.

Symptoms related to cardiopulmonary strain, including shortness of breath and palpitations, were significantly more common in moderate and severe anemia groups compared to normal subjects. Chi-square analysis demonstrated a statistical association between anemia severity and all evaluated symptoms (*p* < 0.001), indicating a clear dose–response relationship between declining hemoglobin levels and symptom burden.

A statistically significant association was observed between dietary intake patterns and anemia severity (Table 5). Regular consumption of iron-rich foods was reported by a higher proportion of participants with normal hemoglobin status (78.7%) compared to those with mild (64.2%), moderate (62.0%), and severe anemia (47.6%) (*p* = 0.002). Similarly, intake of green leafy vegetables and animal-source foods declined progressively with increasing anemia severity. Conversely, the proportion of individuals consuming tea or coffee with meals increased progressively with anemia severity.

**Table 5:** Association between food consumption patterns and anemia severity.

Food consumption pattern	Normal *n* (%)	Mild *n* (%)	Moderate *n* (%)	Severe *n* (%)	χ^2^ value	*p*-value
Regular intake of iron-rich foods	118 (78.7)	176 (64.2)	62 (62.0)	10 (47.6)	14.82	0.002*
Green leafy vegetables ≥3 times/week	104 (69.3)	158 (57.7)	48 (48.0)	7 (33.3)	16.94	<0.001*
Animal-source foods (meat/egg/fish)	96 (64.0)	142 (51.8)	44 (44.0)	6 (28.6)	18.27	<0.001*
Vitamin C–rich fruits daily	110 (73.3)	150 (54.7)	46 (46.0)	8 (38.1)	19.11	<0.001*
Frequent tea/coffee with meals	38 (25.3)	104 (38.0)	52 (52.0)	14 (66.7)	22.86	<0.001*
Regular iron supplementation	54 (36.0)	44 (16.1)	12 (12.0)	2 (9.5)	21.34	<0.001*

*Statistically significant at *p* < 0.05 (Chi-square test).

Vitamin-C rich fruit consumption, which enhances non –heme iron absorption, was significantly more common among normal participants than anemic groups (*p <* 0.001). In contrast, frequent consumption of tea or coffee with meals known inhibitors of iron absorption was significantly higher among participants than with moderate and severe anemia. Regular use of iron supplements was markedly lower among anemic participants, particularly in moderate and severe categories (*p* < 0.001).

Multivariable logistic regression analysis in Table 6 demonstrated that inadequate intake of iron-rich foods, low dietary diversity, frequent intake of iron absorption inhibitors, and lack of iron supplementation were independently associated with higher odds of anemia. The strongest association was observed for non-use of iron supplements (AOR = 2.87, *p* < 0.001) and consumption of tea or coffee with meals (AOR = 2.21, *p* < 0.001).

**Table 6:** Association between food consumption patterns and anemia severity (multivariable analysis).

Dietary factor	AOR	95% CI (lower–upper)	*p*-value
Inadequate intake of iron-rich foods	1.82	1.21–2.73	0.004*
Low consumption of green leafy vegetables	1.67	1.12–2.49	0.011*
Low intake of animal-source foods	1.94	1.28–2.94	0.002*
Infrequent vitamin C–rich fruit intake	1.59	1.06–2.38	0.024*
Tea/coffee consumption with meals	2.21	1.45–3.38	<0.001*
Non-use of iron supplements	2.87	1.72–4.78	<0.001*

*Statistically significant at *p* < 0.05. Outcome variable: Presence of anemia (mild, moderate, or severe vs. normal). Statistical mode: Multivariable logistic regression. Adjusted for: age, residence, stay, marital status.

### Knowledge regarding anemia

Table 7 shows the knowledge responses of the participants. Overall, knowledge about anemia among participants was moderate. Approximately two-thirds of the respondents correctly identified anemia as a condition characterized by a lower than normal number of red blood cells (66.66%) and recognized iron deficiency as a potential cause of anemia (67.21%). Fatigue was acknowledged as a common symptom by 67.57% of participants, while pale skin was correctly identified as a sign of anemia by 68.48%. Awareness of vitamin B12 deficiency as a cause of anemia was observed in 60.65% of respondents.

**Table 7:** Frequency and percentage distribution of knowledge responses on anemia.

Knowledge question	Yes	%	No	%
Anemia-lower than normal number of red blood cells	366	66.66	183	33.33
Lack of iron in the diet lead to anemia	369	67.21	180	32.78
Fatigue-common symptom of anemia	371	67.57	178	32.42
High red blood cell count considered anemia	164	29.87	385	70.12
Heavy menstrual bleeding leads to anemia	301	54.82	248	45.17
Pale skin sign of anemia	376	68.48	173	31.51
Lake of vitamin B12 cause anemia	333	60.65	216	39.34
Anemia without experiencing any noticeable symptoms	230	41.89	319	58.10
Anemia contagious	189	34.42	360	65.57
Chronic diseases lead to anemia	288	52.45	261	47.54

However, important misconceptions were evident. Only 41.89% were aware that anemia can occur without noticeable symptoms, and just 52.45% recognized that chronic diseases can contribute to anemia. Notably, 34.42% incorrectly believed anemia to be contagious, and nearly half of the respondents (45.17%) were unaware of the association between heavy menstrual bleeding and anemia, indicating gaps in understanding of risk factors and disease mechanisms.

### Attitude toward anemia

Attitudinal responses (Table 8) reflected a generally cautious but inconsistent approach toward anemia. While a majority of participants agreed that it is important to follow a doctor’s advice if diagnosed with anemia (77.95%) and believed that young adults should be aware of anemia symptoms (76.68%), fewer respondents’ demonstrated appropriate concern toward early warning signs. Only 56.64% felt that persistent fatigue warrants attention, and 42.80% believed that young adults should not simply accept tiredness as a consequence of busy schedules. 24.22% considered it acceptable to initiate iron supplementation without consulting a doctor, and 35.88% perceived a doctor-recommended blood test for anemia as unnecessary. Additionally, 30.60% felt that anemia mentioned by a friend could be dismissed as a minor issue, suggesting underestimation of the condition’s clinical significance.

**Table 8:** Frequency and percentage distribution of attitude responses on anemia.

Attitude question	Yes	%	No	%
More tired than usual/should I have anemia	205	37.34	344	62.65
Attention to fatigue is required	311	56.64	238	43.35
I ignore paleness in my skin if I do not feel particularly unwell	152	27.68	397	72.31
Is it okay to start taking iron supplements without seeing a doctor	133	24.22	416	75.77
Blood test recommended by a doctor for anemia is unnecessary	197	35.88	352	64.11
Young adults just accept feeling tired due to busy schedules is not consider anemia	235	42.80	314	57.19
Follow doctor’s advice if diagnosed with anemia	428	77.95	121	22.04
Anemia mentioned by a friend should be considered a minor issue	168	30.60	381	69.39
Good idea for young adults to be aware of the symptoms of anemia	421	76.68	128	23.31
Diagnosed with anemia, would I be comfortable discussing it with a healthcare professional	410	74.68	139	25.31

### Practices related to anemia

Preventive and health-seeking practices showed mixed patterns in Table 9. A relatively high proportion of participants reported including iron-rich foods in their regular diet (71.40%) and making efforts to obtain adequate sleep (62.65%). More than half of the respondents sought medical advice when experiencing persistent fatigue (60.10%) and adhered to prescribed treatment when diagnosed with anemia (61.56%).

**Table 9:** Frequency and percentage distribution of practice responses on anemia.

Practice question	Yes	%	No	%
Including iron-rich foods in regular diet	392	71.40	157	28.59
As a female has to track menstrual cycle and flow	327	59.56	125	22.76
Regularly taking a multivitamin or iron supplement	68	12.38	385	70.12
Seek medical advice if experience persistent and unusual fatigue or other concerning symptoms	330	60.10	219	39.89
Diagnosed with anemia, do you consistently take any prescribed medications or supplements	338	61.56	211	38.43
Get regular check-ups with a doctor	123	22.40	338	61.56
Avoid excessive consumption of caffeine or calcium-rich foods with meals?	177	32.24	247	44.99
Donate blood regularly? (Note: Frequent blood donation can sometimes contribute to iron deficiency in susceptible individuals)	84	15.30	406	73.95
An effort to get adequate sleep	344	62.65	205	37.34
Someone with anemia, do you offer them support and understanding?	445	81.05	104	18.94

In contrast, routine preventive practices were suboptimal. Only 22.40% reported regular medical check-ups, and a very small proportion took multivitamin or iron supplements regularly (12.38%). Avoidance of caffeine or calcium-rich foods during meals was reported by just 32.24%. Blood donation practices were also limited, with only 15.30% donating regularly. Encouragingly, a strong social support attitude was observed, as 81.05% reported offering support to individuals known to have anemia.

### Changes in knowledge and attitude related to anemia

Significant improvements were observed in participant’s knowledge regarding anemia symptoms and warning signs. The proportion of participants correctly identifying pale skin as a sign of anemia increased (Table 10) from 66.0% pre-intervention to 84.0% post-intervention, with the change reaching statistical significance (*p* = 0.0039). Similarly, awareness that fatigue is a common symptom of anemia improved from 58.0 to 74.0%, also demonstrating a significant increase (*p* = 0.0078). A favorable shift was noted in attitude-based responses. Agreement with the statement “I should ignore paleness in my skin if I do not feel unwell” decreased substantially from 42.0 to 22.0% (*p* = 0.0063), reflecting improved health-seeking awareness. Additionally, responses to the statement “if I feel slightly more tired than usual, I should immediately worry” declined from 51.0 to 32.7% (*p* = 0.0078), indicating a shift toward more balanced and informed symptom interpretation rather than excessive concern.

**Table 10:** Pre- and post-intervention results (knowledge and attitude on anemia).

Domain	Variable/statement	Pre (%)	Post (%)	Change (%)	*p*-value	Significance
Knowledge	Identification of pale skin as a sign of anemia	66.0	84.0	18.0	0.0039	Significant
Knowledge	Awareness that fatigue is a symptom of anemia	58.0	74.0	16.0	0.0078	Significant
Attitude	“I should ignore paleness if I do not feel unwell”	42.0	22.0	20.0	0.0063	Significant (Improved)
Attitude	“If I feel slightly tired, I should immediately worry”	51.0	32.7	18.3	0.0078	Significant (Improved)

## Discussion

The predominance of participants in the 20–22 year age group is consistent with the study population being largely composed of young adults, a demographic recognized as nutritionally vulnerable due to increased physiological demands, lifestyle transitions, and irregular dietary practices. Similar age distributions have been reported in studies conducted among college-going populations, where anemia and micronutrient deficiencies remain prevalent public health concerns (World Health Organization, 2011a; World Health Organization, 2011b; Kassebaum et al., 2014).

The overwhelmingly unmarried status observed in the present study aligns with the age structure of the cohort and mirrors findings from comparable institutional-based studies. Marital status may indirectly influence nutritional status through dietary habits and health-seeking behavior, although its impact is often minimal in younger populations (Bentley and Griffiths, 2003).

The nearly equal representation of urban and rural participants strengthens the generalizability of the findings, as anemia risk is influenced by both urban lifestyle factors and rural socioeconomic constraints. A study by Balarajan et al. (2011) has demonstrated that while rural populations often face nutritional inadequacies, urban populations are increasingly affected by poor dietary diversity and sedentary lifestyles, contributing to anemia risk across both settings (Balarajan et al., 2011).

The higher proportion of hostellers observed in the study is noteworthy, as hostel living has been associated with irregular meal patterns, limited dietary choices, and increased reliance on processed foods. These factors may adversely affect micronutrient intake, particularly iron, thereby increasing susceptibility to anemia (Gautam et al., 2023).

The blood group distribution, with B + and O + being the most common, reflects patterns reported in several Indian and south Asian population studies. Although blood group itself is not a direct determinant of anemia, understanding its distribution is useful for clinical and transfusion-related considerations and for contextualizing hematological findings (Shah et al., 2022).

An important finding of this study is the significant association between AB+ blood group and anemia. Although the biological mechanisms linking ABO blood groups and anemia are not fully understood, variations in hemoglobin levels, erythrocyte membrane properties, and susceptibility to nutritional deficiencies have been suggested as possible explanations (Al-Khikani and Ayit, 2025). The persistence of this association after multivariable adjustment underscores the need for further research to explore genetic and hematological factors influencing anemia risk.

Blood group distribution mirrored patterns reported in south Asian populations, with B + and O + being most prevalent (Agrawal et al., 2014). Although no strong visual association between blood group and anemia severity emerged, existing literature suggests that blood group itself is not a primary determinant of anemia risk, with nutritional, menstrual, and inflammatory factors playing more dominant roles (Beutler and Waalen, 2006).

Overall, the demographic characteristics of the study population highlight a group that is biologically and behaviorally predisposed to anemia, underscoring the importance of targeted screening, nutrition education, and preventive interventions among young adults.

The present study highlights a high burden of anemia among young adults, with mild anemia being the predominant from across all demographic categories. This finding aligns with global and regional evidence indicating that mild anemia is highly prevalent in young populations and often remains under-recognized due to subtle or absent symptoms (World Health Organization, 2011a; World Health Organization, 2011b; Kassebaum et al., 2014).

The observed age-wise distribution suggests that individuals in the early twenties are particularly vulnerable, possibly due to increased academic stress, irregular dietary habits, and inadequate intake of iron-rich foods. Similar trends have been reported among college-going populations, where lifestyle factors and nutritional inadequacies contribute significantly to anemia risk (Balarajan et al., 2011).

The comparable prevalence of anemia between rural and urban participants suggests that anemia is no longer confined to rural or socioeconomically disadvantaged settings. Urbanization-related dietary transitions, increased consumption of processed foods, and sedentary lifestyle may explain the persistence of anemia in urban populations (Bentley and Griffiths, 2003).

Hostellers exhibited a higher prevalence of anemia compared to day scholars, which may be attributed to dependence on institutional food services, limited dietary diversity, and irregular meal timing. A study by Hani et al. (2026) has similarly reported higher anemia prevalence among individuals residing in hostels or shared accommodations (Hani et al., 2026).

Overall, the findings emphasize that anemia among young adults is multifactorial and influenced by lifestyle, living conditions and biological factors. Early screening, nutrition education, and targeted interventions are essential to prevent progression to moderate or severe anemia and to improve long-term health outcomes.

The present findings provide important insights into the demographic profile and hematological status of young adults, a population often considered clinically healthy but increasingly recognized as vulnerable to nutritional deficiencies. The mean hemoglobin level observed in this study falls below the World Health Organization cut-off for non-pregnant adults, indicating a substantial burden of anemia or borderline anemia within the cohort. Similar trends have been reported in population-based studies among adolescents and young adults, where iron deficiency remains the predominant contributor to reduced hemoglobin levels (Kassebaum et al., 2014; World Health Organization, 2020).

The observed PCV, MCV, and MCH values suggest a predominance of normocytic to mildly microcytic red cell morphology, which is commonly associated with iron deficiency anemia and anemia of chronic inflammation. This aligns with earlier reports indicating that young adults frequently exhibit early hematological changes before overt clinical symptoms become evident (de Benoist et al., 2008). The relatively wide range in MCV and MCH values further highlights the heterogeneity of anemiaetiology within the population.

White blood cell and platelet parameters were largely within normal reference ranges, suggesting the absence of acute infection or hematological malignancy in most participants. This supports the likelihood that the reduced hemoglobin levels are primarily linked to nutritional and lifestyle factors rather than systemic disease. Moderate energy and sleep quality scores observed in the study may also play an indirect role, as inadequate sleep and fatigue have been associated with poorer nutritional status and impaired hematopoiesis (Cappuccio et al., 2011).

Taken together, these findings underscore the importance of routine hematological screening and early nutritional interventions among young adults. Addressing subclinical anemia at this stage may prevent progression to more severe forms and improve overall health, academic performance, and quality of life. Public health strategies focusing on dietary education, regular health check-ups, and anemia awareness are therefore strongly warranted.

The findings demonstrate a high burden of anemia among apparently healthy young adults, despite relatively normal anthropometric profiles. This highlights the limitation of using body size alone as an indicator of nutritional or hematological health and aligns with global evidence that iron deficiency and anemia frequently coexist with normal body mass indices, particularly in adolescents and young adults (Kassebaum et al., 2014; World Health Organization, 2020). The predominance of mild and moderate anemia suggests a chronic, subclinical pattern that may remain undetected without routine screening.

The clear gradation of hemoglobin values across anemia severity categories reinforces the biological validity of the classification and reflects expected hematological trends. Strong correlations between hemoglobin, PCV, and red cell indices are consistent with iron-deficiency-driven erythropoietic changes, where reduced hemoglobin synthesis leads to microcytic, hypochromic red cells (Camaschella et al., 2016). The modest inverse association between platelet count and hemoglobin observed in this study has been reported and may reflect reactive thrombocytosis in iron deficiency states (Park et al., 2013).

Importantly, the observed improvements in knowledge, attitude, and practice indicators suggest that targeted awareness efforts can effectively correct misconceptions and promote positive health behaviors. Increased recognition that anemia may be asymptomatic and that persistent fatigue warrants medical attention is particularly relevant, as delayed diagnosis is a major contributor to disease progression in young population (Pasricha et al., 2021). However, despite improved awareness, the continued high prevalence of anemia indicates the knowledge alone may be insufficient to achieve optimal hematological outcomes. Structural factors such as dietary quality, access to iron-rich foods, menstrual health management, and adherence to supplementation likely influence the translation of awareness into sustained practice.

Overall, these findings underscore the need for integrated interventions combining routine screening, nutrition-focused strategies, and sustained behavior-change communication. Addressing anemia during young adulthood is critical, as untreated deficiency at this stage can impair physical performance, cognitive function, and future reproductive health, thereby extending its impact across the life course.

The findings of this study highlight a strong and graded association between anemia severity and the manifestation of classical clinical symptoms. The predominance of fatigue even in mild anemia underscores the functional impact of early hemoglobin depletion, which is often underestimated in young adults. Similar observations have been documented in epidemiological studies where mild anemia was associated with reduced physical performance and cognitive efficiency (World Health Organization, 2011a; World Health Organization, 2011b; Kassebaum et al., 2014).

The sharp increase in pallor, dizziness, and breathlessness in moderate and severe anemia reflects impaired oxygen delivery and compensatory cardiovascular responses. These findings are consistent with earlier reports emphasizing that symptom intensity escalates as hemoglobin levels decline. The significant association between anemia severity and cardiopulmonary symptoms further reinforces the need for early detection to prevent progression to clinically debilitating stages (Obeagu et al., 2023; Volchkova and Subkhankulova, 2022).

Overall, the results emphasize that anemia should not be viewed solely as a laboratory abnormality but as a condition with measurable clinical consequences. Routine screening, symptom awareness, and timely intervention remain critical, particularly in young adult populations where anemia often remains undiagnosed despite substantial symptomatology (Kumar et al., 2025).

The present findings demonstrate a clear relationship between dietary practices and anemia severity. Participants with normal hemoglobin levels reported significantly better dietary patterns, including higher intake of iron-rich foods, green leafy vegetables, animal-source foods, and vitamin-C rich fruits. These dietary behaviors are well-established protective factors against nutritional anemia and support optimal iron bioavailability (Balarajan et al., 2011; Bhatnagar and Padilla-Zakour, 2021).

Conversely, the higher prevalence of tea and coffee consumption during meals among moderately and severely anemic participants likely contributes to impaired iron absorption due to polyphenolic compounds. The low prevalence of iron supplementation among anemic individuals highlights gaps in awareness, adherence, or anemia prevention and control programs (Kolarš et al., 2025).

This study provides clear evidence that dietary practices play a critical role in determining anemia status among young adults. Individuals with normal hemoglobin levels demonstrated significantly healthier dietary patterns, including greater intake of iron-rich and iron-enhancing foods. In contrast, anemic participants particularly those with moderate and severe anemia exhibited lower dietary quality and higher exposure to inhibitors of iron absorption.

The strong association between tea or coffee consumption during meals and anemia severity is consistent with established evidence on the inhibitory effects of polyphenols on non-heme iron absorption. Similarly, the reduced intake of animal-source foods among anemic individuals highlights the importance of heme iron sources, which have superior bioavailability compared to plant-based iron.

The multivariable findings underscore that poor dietary intake remains an independent predictor of anemia even after controlling for demographic factors. These results reinforce the need for targeted nutrition education, dietary diversification strategies, and appropriate supplementation programs, particularly for young women who are biologically vulnerable to iron deficiency.

Overall, the findings support nutrition – focused preventive approach and align with global recommendations emphasizing diet-based interventions as a cornerstone of anemia control strategies.

The present study highlights moderate levels of knowledge regarding anemia among young adults, with relatively better awareness of basic definitions and common symptoms, but notable deficiencies in understanding asymptomatic anemia, chronic disease associations, and menstrual-related risks. Similar patterns have been reported in studies among university students and young populations, where general awareness exists but deeper clinical understanding remains limited (Kassebaum et al., 2014; World Health Organization, 2020).

Attitudinal findings indicate that although most participants value medical guidance and symptom awareness, a substantial proportion underestimate the seriousness of anemia or normalize fatigue due to lifestyle factors. This tendency to trivialize symptoms has been documented in previous studies, where fatigue is often attributed to academic or occupational stress rather than underlying nutritional deficiencies (De Benoist et al., 2008). Misconceptions regarding self-medication with iron supplements are particularly concerning, as inappropriate supplementation may mask underlying conditions or cause adverse effects.

Practice related findings reveal a gap between knowledge and behavior. While dietary inclusion of iron – rich foods was relatively common, regular health monitoring and supplement use were poor. This discrepancy mirrors earlier reports indicating that awareness alone does not consistently translate into preventive practices. The low rate of routine check-ups further underscores the need for structured screening programs and targeted health education initiatives.

Collectively, these findings emphasize the importance of comprehensive anemia education programs that not only improve knowledge but also address attitudes and promote sustainable health practices. Integrating anemia awareness into campus-based health services and primary care outreach may help bridge existing gaps and reduce the burden of anemia among young adults.

The present study demonstrates that the anemia app was effective in improving sleep quality, hemoglobin concentration, and anemia – related knowledge and attitudes, although its effect on perceived energy levels was limited.

The significant improvement in sleep quality aligns with previous findings that nutritional and health education interventions can positively influence sleep through improved physiological regulation and symptom awareness (Szymczak et al., 2025). Improved sleep quality may also indirectly support hematological recovery by reducing inflammatory stress and enhancing metabolic efficiency.

Although the increase in hemoglobin levels was numerically small, its statistical significance suggests a consistent improvement across participants, which is clinically relevant in populations with low baseline hemoglobin. Similar modest yet significant hemoglobin gains have been reported in short-term or low-intensity interventions, particularly when baseline anemia is prevalent (Balarajan et al., 2011; World Health Organization, 2020).

The absence of significant change in energy levels may be explained by the multifactorial nature of fatigue, which is influenced not only by hemoglobin concentration but also by sleep, psychological stress, physical work load, and micronutrient status. Short intervention duration and subjective measurement tools may further limit detectable changes in perceived energy (Pasricha et al., 2021).

Improvements in knowledge and attitudes toward anemia were among the most pronounced outcomes. Increased recognition of key symptoms such as pallor and fatigue, along with reduced tendencies to ignore warning signs, reflects effective health communication. Knowledge gains of this nature are critical, as awareness is a strong predictor of early diagnosis and adherence to preventive strategies (Aidoo, 2025).

Overall, the findings suggest that while physiological changes may require longer or more intensive interventions, behavioral and cognitive outcomes respond rapidly and robustly, reinforcing the value of structured education-based programs in anemia prevention and management.

### About the AI application

The developed AI-based application is designed to assist in the screening and preliminary classification of anemia based on input parameters such as hemoglobin levels and related assessment criteria. The primary objective of the application is to provide a quick, accessible, and supportive diagnostic tool that can help identify individuals who may require further medical evaluation.

The application uses a rule-based and pattern-recognition approach to categorize anemia into Normal, Mild, Moderate, and Severe levels. By analyzing the input data, the AI system generates an assessment result, which is then compared with standard hemoglobin classification to evaluate its accuracy and performance.

In the present evaluation, the AI application achieved an overall accuracy of approximately 71%. It demonstrated relatively high sensitivity in detecting anemia cases, particularly moderate and severe forms, while showing moderate limitations in correctly classifying normal cases. This pattern is consistent with several existing digital health screening tools, which are often optimized for sensitivity to minimize missed diagnoses in public health settings.

Similar findings have been reported in AI-enabled anemia screening applications such as HemaApp, a smartphone-based tool that estimates hemoglobin levels using image analysis. Studies have shown that while such applications are effective for identifying anemia, confirmatory laboratory testing remains necessary for accurate diagnosis (Mannino et al., 2018). Likewise, AnemoCheck Mobile has demonstrated utility as a low-cost point-of-care screening tool, particularly for detecting moderate to severe anemia, though with reduced precision in identifying normal hemoglobin levels (Tyburski et al., 2014).

Furthermore, broader artificial intelligence platforms such as IBM Watson Health have illustrated the potential of AI in enhancing clinical decision-making, particularly in early detection and risk stratification. These systems reinforce the concept that AI tools should function as supportive screening aids rather than replacements for standard diagnostic methods (Esteva et al., 2019).

The AI application developed in this study offers several practical advantages:

- Rapid preliminary assessment.
- Reduction in manual classification errors.
- Support for large-scale community-level screening.
- Ease of use in low-resource and field settings.

These benefits align with global efforts to integrate digital health tools into primary healthcare systems, especially in resource-constrained environments.

With further refinement, including the use of larger and more diverse datasets, improved algorithm design, and incorporation of additional clinical indicators, the diagnostic performance of the application can be enhanced. Future developments may also include automated reporting systems, integration with electronic health records, and real-time data monitoring, thereby strengthening its utility in public health surveillance and anemia control programs.

### Implications to practice

The findings of this study have important implications for both clinical practice and public health interventions. The observed improvement in participants’ knowledge regarding anemia symptoms and warning signs indicates that structured educational interventions can effectively enhance awareness. This suggests that incorporating regular health education programs into community and institutional settings can play a key role in early recognition and prevention of anemia. From a clinical perspective, increased awareness of symptoms such as fatigue and pale skin can encourage individuals to seek timely medical attention, thereby facilitating early diagnosis and treatment. Healthcare providers, especially at the primary care level, can integrate simple screening and counseling strategies into routine services to identify at-risk populations more efficiently.

The integration of artificial intelligence (AI) in the study further highlights its potential in strengthening anemia management. AI-based tools can support:

- Early detection through predictive risk assessment
- Efficient screening of large populations
- Decision support for healthcare professionals

Such technologies can be particularly valuable in resource-limited settings, where access to specialized care is limited. AI-driven models can assist frontline health workers in identifying high-risk individuals and prioritizing interventions.

Additionally, the identification of key determinants of anemia provides actionable insights for targeted interventions. Public health programs can focus on high-risk groups and address modifiable risk factors such as nutrition, hygiene, and health-seeking behavior.

Overall, the study supports a combined approach involving health education, routine screening, and AI-assisted decision-making to improve anemia prevention and management. Implementing these strategies in real-world settings can contribute to reducing the burden of anemia and improving population health outcomes.

### Limitations of the study

The main limitations of the study include relatively small dataset size, class imbalance (especially fewer severe cases), reliance on questionnaire based features instead of clinical biomarkers like hemoglobin levels and lack of external validation. A significant proportion of participants were classified as anemic (72.48%), which may have biased the model toward predicting the majority class. The dataset may not represent broader populations due to geographic and demographic constraints. Label encoding may impose artificial ordinal relationships among categorical variables. Important biomarkers such as serum ferritin and iron levels were not included. Due to funding limitations, study conducted on the Hb and hematological parameters, future work can improve accuracy by including larger datasets, laboratory test parameters and advanced optimization techniques.

## Conclusion

This study demonstrates that anemia remains a significant public health concern among young adults, with dietary factors emerging as key modifiable determinants of both its occurrence and severity. The strong association between poor dietary intake, reduced iron bioavailability, and increasing anemia severity underscores the importance of early nutritional interventions. The presence of symptoms even in mild anemia reinforces the need for routine screening and heightened awareness to prevent progression to more severe stages.

Targeted nutrition education focusing on improved dietary diversity, increased consumption of iron –rich and iron-enhancing foods, and appropriate use of iron supplementation is essential. Additionally, behavioral modifications such as reducing tea or coffee intake during meals may contribute meaning fully to anemia prevention. Integrating dietary counseling with regular health assessments can play a crucial role in reducing the burden of anemia and improving overall health outcomes in young adult populations.

The AI-based anemia assessment application represents a significant step forward in leveraging digital health technologies for scalable anemia screening. The tool demonstrated moderate accuracy in classifying anemia status when compared with standard hemoglobin measurements in our evaluation, highlighting its potential as a supportive screening aid. Its ease of use, rapid assessment capability, and potential to reach underserved populations underline the value of integrating artificial intelligence into preventive health strategies. Although it is not intended to replace clinical diagnosis, the application offers valuable preliminary insights that can prompt timely medical consultation and intervention. With ongoing improvements in algorithm precision and incorporation of broader clinical data, this AI application could play a pivotal role in enhancing early detection, monitoring, and public health outcomes related to anemia.

## Data Availability

The raw data supporting the conclusions of this article will be made available by the authors, without undue reservation.
